# Association between serum indoxyl sulfate levels with carotid-femoral pulse wave velocity in patients with chronic kidney disease

**DOI:** 10.1080/0886022X.2021.1921797

**Published:** 2021-05-03

**Authors:** Sheng-Chao Wang, Yu-Hsien Lai, Chin-Hung Liu, Chih-Hsien Wang, Bang-Gee Hsu, Jen-Pi Tsai

**Affiliations:** a Division of Nephrology, Hualien Tzu Chi Hospital, Buddhist Tzu Chi Medical Foundation, Hualien, Taiwan; b School of Medicine, Tzu Chi University, Hualien, Taiwan; c Department of Pharmacology, Tzu Chi University, Hualien, Taiwan; d Cardiovascular Research Centre, Hualien Tzu Chi Hospital, Buddhist Tzu Chi Medical Foundation, Taiwan; e Division of Nephrology, Department of Internal Medicine, Dalin Tzu Chi Hospital, Buddhist Tzu Chi Medical Foundation, Chiayi, Taiwan

**Keywords:** Arterial stiffness, carotid-femoral pulse wave velocity, chronic kidney disease, indoxyl sulfate

## Abstract

**Background:**

The role of indoxyl sulfate (IS), an important protein-bound uremic toxin, in arterial stiffness (AS) in patients with chronic kidney disease (CKD) is unclear.

**Materials and methods:**

We investigated the association between serum IS levels and AS in a cross-sectional study of 155 patients with CKD. Patients in the AS group was defined as carotid-femoral pulse wave velocity (cfPWV) value >10 m/s measured by a validated tonometry system (SphygmoCor), while values ≤10 m/s were regarded as without AS group Serum IS was measured by liquid chromatography–mass spectrometry analysis.

**Results:**

Of these CKD patients, AS was present in 51 (32.9%) patients, who were older, had a higher rate of diabetes, higher systolic blood pressure (SBP), and higher IS levels compared to those without AS. By multivariable logistic regression analysis, IS (adjusted odds ratio [aOR] 1.436, 95% confidence interval [CI] 1.085–1.901, *p* = 0.011), age (aOR 1.058, 95% CI 1.021–1.097, *p* = 0.002), and SBP (aOR 1.019, 95%CI 1.000–1.038, *p* = 0.049) were independent predictors of AS. By multivariable stepwise linear regression analysis, logarithmically transformed IS, age, DM, and SBP were significantly correlated with cfPWV. The area under the receiver-operating characteristic curve for serum log-IS was 0.677 (95%CI 0.598–0.750, *p* = 0.0001) to predict the development of AS in patients with CKD.

**Conclusion:**

These finding demonstrate that in addition to older and higher SBP, a high serum IS level is a significant biomarker associated with AS in patients with CKD.

## Introduction

The incidence of cardiovascular disease (CVD) is significantly higher in patients with chronic kidney disease (CKD) than in those without CKD. CVD in patients with CKD is associated with poor morbidity and mortality due to traditional risk factors including age, hypertension, and diabetes mellitus (DM) as well as CKD-specific factors such as arterial stiffness (AS) [1,2]. AS is caused by irreversible changes to the vascular structure, including dysregulation of elastin and collagen due to oxidative stress and inflammation, which result in impaired perfusion of vital organs [3,4]. Several studies have identified age, hypertension, DM, and abnormal renal function as risk factors for AS [3,5–10]. Importantly, indoxyl sulfate (IS), originally identified as a gut-derived, protein-bound uremic toxin, accumulates with declining renal function and contributes to the deterioration of kidney function through several mechanisms. For example, several lines of evidence from *in vitro* and *in vivo* studies have demonstrated that IS induces renal tubular damage and tubulointerstitial fibrosis through oxidative stress, inflammation, and trans-differentiation of renal tubular cells *via* the upregulation of intra-renal renin-angiotensin-aldosterone or mitogen-activated protein kinase pathway, which culminate in renal fibrosis [11–13]. In addition, IS can augment oxidative stress, increase endothelial microparticle generation, impair endothelial cell repair, and induce vascular smooth muscle cell proliferation, ultimately resulting in aortic calcification and AS, which can lead to increased overall and cardiovascular mortality in patients with CKD [14].

While IS may play a role in vascular dysfunction and can induce further cardiovascular events in patients with CKD, there are no reliable circulating biomarkers for vascular dysfunction. Therefore, we aimed to identify the predictors for the development of AS in patients with CKD and specifically examined the potential association between serum IS levels and AS in these patients.

## Materials and methods

### Participants

In this cross-sectional study conducted at a single hospital. After the approval from The Protection of the Human Subjects Institutional Review Board of Tzu-Chi University and Hospital (IRB108-96-B) and receiving the informed consents from all patients from January and December 2016, there were 155 out of 180 CKD patients enrolled because we excluded 25 patients due to missing data (15), acute infection (5), acute myocardial infarction or heart failure (4) and malignancy (1). Hypertension and DM were diagnosed according to SBP or DBP ≥ 140/90 mmHg or fasting plasma glucose level of ≥126 mg/dL as well as usage of relevant medications. Body mass index was measured as (BW)(kg)/(BH) [2](m^2^).

### Biochemical analysis

After obtaining blood samples, serum biochemical analysis was examined by an auto-analyzer (Siemens Advia 1800, Siemens Healthcare, Henkestr, Germany). The estimated GFR was calculated by the Chronic Kidney Disease Epidemiology Collaboration equation [1]: 141 X min(S_cr_/*κ*, 1) ^α^ X max(Scr/*κ*, 1)^−1.209^ × 0.993^Age^ X 1.018 [if female] X 1.159 [if African American]; where S_cr_ is serum creatinine, *κ* is 0.7 for females and 0.9 for males, *α* is −0.329 for females and −0.411 for males, min indicates the minimum of S_cr_/*κ* or 1, and max indicates the maximum of S_cr_/*κ* or 1. Patients were staged according to 2012 Kidney Disease: Improving Global Outcomes Chronic Kidney Disease Guideline [1].

### Determination of serum total is levels

Like our previous study, a Waters e2695 high-performance liquid chromatography system comprising a single quadrupole mass spectrometer with proper performance as well as adequate relative standard deviation of intra- and inter-day precision (within ±15%) was used to measure serum total IS levels (ACQUITY QDa, Waters Corporation, Milford, MA, USA) [15,16].

### Measurement of carotid-femoral pulse wave velocity

AS previously studies [17–19], we used an applanation tonometer (SphygmoCor, AtCor Medical, Australia) to calculate carotid-femoral pulse wave velocity (cfPWV) by recording the pulse waves of carotid and femoral arteries in series synchronously with an electrocardiogram as a timing reference. Calculation of cfPWV was as distance divided by transit time of propagation wave between sternal notch (N) and femoral artery (F) and sternal notch and carotid artery (C):　ΔD/Δt = Distance (N – F) – Distance (N – C) /time interval (N-F) – time interval (N-C). Patients with or without AS were defined according to cfPWV >10 m/s or ≤10 m/s, individually [20].

### Statistical analysis

Continuous variables were analyzed by Student’s independent *t* test or the Mann-Whitney *U* test (two-tailed) and expressed as means ± standard deviation or medians with IQR according to the Kolmogorov–Smirnov test. Categorical variables were analyzed by chi-square test and expressed as numbers and percentage. Values of IS in different stage of CKD was analyzed by one-way analysis of variance. Log-transformed those skewed continuous variable were applied to achieve normal distribution and were used for linear regression analysis. To explore the correlation between variables and cfPWV and possible probabilities for the diagnosis of AS of CKD patients, multivariate linear and logistic regression analyses were applied. Additionally, the relationship between eGFR and IS values was analyzed by linear correlation analysis. To determine the optimal serum IS value to predict AS in CKD patient, a receiver-operating characteristic curve (ROC) analysis was used to calculate the area under the curve (AUC). Data were analyzed using the SPSS for Windows software (version 19.0; SPSS, Chicago, IL, USA).

## Results

Table 1 shows the clinical characteristics of the study cohort of 155 patients with CKD. The cohort comprised 66 (42.6%) and 122 (78.7%) patients with DM and hypertension, respectively. There were 51 (32.9%) patients who were diagnosed to have AS. Compared to patients without AS, patients with AS were older and had more DM (*p* < 0.001 and = 0.030, respectively). Additionally, the patients in the AS group had higher systolic blood pressure (*p* = 0.004), higher IS level (1.53 μg/mL [interquartile range (IQR) 0.70–3.68 μg/mL] vs. 0.88 μg/mL [IQR 0.43–1.76 μg/mL], *p* < 0.001), and lower estimated glomerular filtration rate (*p* = 0.014) compared to those without AS. There were no significant differences in sex, rate of hypertension, and use of medications between the two patient groups.

**Table 1.** Baseline characteristics of CKD patients with or without AS.

Characteristics	All patients (*n* = 155)	Group without AS (*n* = 104)	Group with AS (*n* = 51)	*p*
Age (years)	66.65 ± 13.51	63.75 ± 13.39	72.55 ± 11.81	<0.001*
Female, *n* (%)	73 (47.1)	51 (49.0)	22 (43.1)	0.489
BMI (kg/m^2^)	26.29 ± 4.39	26.30 ± 4.78	26.25 ± 3.53	0.944
<18.5 (underweight), *n*	4	4	0	0.487
18.5–25, *n*	57	37	20	
25–30 (overweight), *n*	66	43	23	
>30 (obesity), *n*	28	20	8	
cfPWV (m/s)	9.34 ± 2.89	7.68 ± 1.46	12.75 ± 1.96	<0.001*
DM, *n* (%)	66 (42.6)	38 (38.5)	28 (54.9)	0.030*
HTN, *n* (%)	122 (78.7)	85 (81.7)	37 (72.5)	0.189
GN, *n* (%)	40 (25.8)	30 (28.8)	10 (19.6)	0.217
Smoking, *n* (%)	16 (10.3)	10 (9.6)	6 (11.8)	0.170
SBP (mmHg)	146.75 ± 21.95	143.18 ± 20.71	154.02 ± 22.81	0.004*
DBP (mmHg)	83.48 ± 12.75	82.94 ± 11.20	84.59 ± 14.21	0.434
BUN (mg/dL)	28.0 (22.0–40.0)	26.0 (20.0–38.0)	31.0 (24.0–41.0)	0.068
Creatinine (mg/dL)	1.60 (1.30–2.20)	1.60 (1.20–2.28)	1.70 (1.40–2.20)	0.053
eGFR (mL/min)	40.40 ± 21.86	43.41 ± 23.54	34.26 ± 16.52	0.014*
IS (μg/mL)	1.06 (0.55–2.17)	0.88 (0.43–1.76)	1.53 (0.70–3.68)	<0.001*
TCH (mg/dL)	160.68 ± 40.49	159.48 ± 44.53	163.12 ± 30.91	0.601
TG (mg/dL)	121.0 (91.0–169.0)	118.50 (91.25–168.75)	168.75 (122.91–170.0)	0.971
LDL-C (mg/dL)	89.15 ± 34.59	88.07 ± 37.15	91.33 ± 28.91	0.585
Fasting glucose (mg/dL)	106.0 (97.0–140.0)	104.0 (97.0–137.25)	109.0 (97.0–157.0)	0.400
Total calcium (mg/dL)	9.06 ± 0.53	9.05 ± 0.52	9.08 ± 0.56	0.740
Phosphorus (mg/dL)	3.69 ± 0.68	3.69 ± 0.62	3.69 ± 0.72	0.995
Total calcium (mg/dL)	9.06 ± 0.53	9.05 ± 0.52	9.08 ± 0.56	0.740
ARB use, *n* (%)	75 (48.4)	53 (51.0)	22 (43.1)	0.360
β-blocker use, *n* (%)	42 (27.1)	32 (30.8)	10 (19.6)	0.142
α-blocker use, *n* (%)	35 (22.6)	21 (20.2)	14 (27.5)	0.310
CCB use, *n* (%)	69 (44.5)	47 (45.2)	22 (43.1)	0.809
Statin use, *n* (%)	75 (48.4)	50 (48.1)	25 (49.0)	0.912
Fibrate use, *n* (%)	15 (9.7)	11 (10.6)	4 (7.8)	0.589
CKD stage 1, *n* (%)	8 (5.2)	7 (6.7)	1 (2.0)	0.294
CKD stage 2, *n* (%)	14 (9.0)	12 (11.5)	2 (3.9)	
CKD stage 3, *n* (%)	75 (48.4)	50 (48.1)	25 (49.0)	
CKD stage 4, *n* (%)	41 (26.5)	25 (24.0)	16 (31.4)	
CKD stage 5, *n* (%)	17 (11.0)	10 (9.6)	7 (13.7)	

Values for continuous variables are given as mean ± standard deviation or median and interquartile range and tested by Student’s *t*-test or Mann–Whitney *U* test according to whether normally distribute; Categorical values are presented as number (%) and analysis was done using the chi-square test.

DM: diabetes mellitus; HTN: hypertension; GN: glomerulonephritis; TG: triglyceride; TCH: total cholesterol; CKD: chronic kidney disease; IS: indoxyl sulfate; cfPWV: carotid–femoral pulse wave velocity; SBP: systolic blood pressure; DBP: diastolic blood pressure; LDL-C: low-density lipoprotein cholesterol; eGFR: estimated glomerular filtration rate; ARB: angiotensin-receptor blocker; CCB: calcium channel blocker.

*p < 0.05 was considered statistically significant.

Multivariable logistic regression analysis performed after adjusting for factors including age, DM, SBP, eGFR, overweight, obesity, fasting glucose and IS, IS (adjusted odds ratio [aOR] 1.436, 95% confidence interval [CI] 1.085–1.901, *p* = 0.011), SBP (aOR 1.019, 95% CI 1.000–1.038, *p* = 0.049), and age (OR 1.058, 95% CI 1.021–1.097, *p* = 0.002) were significant and independent predictive factors for AS (Table 2).

**Table 2.** Factors predicted the development of AS among CKD patients.

Variables	OR	95% CI	*p*	aOR	95% C.I.	*p*
IS, 1 μg/mL	1.416	1.159–1.729	0.001	1.436	1.085–1.901	0.011*
Age, 1 year	1.057	1.026–1.088	<0.001	1.058	1.021–1.097	0.002*
Gender, Female	0.788	0.402–1.576	0.490			
HTN, present	0.591	0.268–1.303	0.192			
DM, present	2.114	1.070–4.177	0.031	1.415	0.545–3.674	0.475
Smoking, present	1.253	0.429–3.664	0.680			
Overweight (BMI 25–30 kg/m^2^)	0.990	0.471–2.081	0.978	0.866	0.359–2.089	0.749
Obesity (BM*I* > 30 kg/m^2^)	0.740	0.277–1.980	0.549	1.040	0.340–3.184	0.945
SBP, 1 mmHg	1.024	1.007–1.041	0.005	1.019	1.000–1.038	0.049*
DBP, 1 mmHg	1.011	0.984–1.039	0.431			
TCH, 1 mg/dL	1.002	0.994–1.011	0.599			
TG, 1 mg/dL	1.001	0.999–1.003	0.311			
Fasting glucose, 1 mg/dL	1.008	1.000–1.015	0.045	1.005	0.995–1.015	0.339
Total calcium, 1 mg/dL	1.114	0.592–2.098	0.738			
Phosphorus, 1 mg/dL	1.002	0.597–1.679	0.995			
eGFR, 1 mL/min	0.978	0.960–0.996	0.016	1.013	0.987–1.040	0.321

Analysis data was done using the multivariate logistic regression analysis (adopted factors: diabetes mellitus, age, systolic blood pressure, eGFR, overweight, obesity, fasting glucose and indoxyl sulfate).

eGFR: estimated glomerular filtration rate.

*p < 0.05 was considered statistically significant.

By simple linear regression analysis, carotid-femoral pulse wave velocity (cfPWV) was significantly and positively correlated with age, DM, SBP, logarithmically transformed (log)-IS (log-IS), log-creatinine, and log-blood urea nitrogen (BUN) and was negatively correlated with eGFR. The multivariable stepwise linear regression analysis performed after adjusting for factors including DM, age, SBP, eGFR, and log-IS, cfPWV was significantly correlated with age (*β* = 0.267, adjusted *R*^2^ change = 0.052, *p* < 0.001), SBP (*β* = 0.283, adjusted *R*^2^ change = 0.094, *p* < 0.001), DM (β = 0.177, adjusted *R*^2^ change = 0.026, *p* = 0.011), and log-IS level (β = 0.217, adjusted R^2^ change = 0.133, *p* = 0.003) in patients with CKD (Table 3).

**Table 3.** Correlation between cfPWV and clinical variables among the CKD patients.

Variables	Carotid-femoral pulse wave velocity (m/s)				
	Simple regression		Multivariable regression		
	*r*	*p*	Beta	Adjusted *R*^2^ change	*p*
Female	−0.123	0.127	–	–	–
Diabetes mellitus	0.236	0.003*	0.177	0.026	0.011*
Hypertension	−0.041	0.611	–	–	–
Glomerulonephritis	−0.093	0.250	–	–	–
Smoking	−0.031	0.702			
Age (years)	0.365	<0.001*	0.267	0.052	<0.001*
Body mass index (kg/m^2^)	0.103	0.201	–	–	–
SBP (mmHg)	0.365	<0.001*	0.283	0.094	<0.001*
DBP (mmHg)	0.139	0.086	–	–	–
TCH (mg/dL)	−0.087	0.283	–	–	–
Log-Triglyceride (mg/dL)	0.018	0.821	–	–	–
LDL-C (mg/dL)	−0.106	0.190	–	–	–
Log-Glucose (mg/dL)	0.119	0.140	–	–	–
Log-BUN (mg/dL)	0.203	0.011*	–	–	–
Log-Creatinine (mg/dL)	0.245	0.002*	–	–	–
eGFR (mL/min)	−0.284	<0.001*	–	–	–
Total calcium (mg/dL)	0.025	0.754	–	–	–
Phosphorus (mg/dL)	0.002	0.977	–	–	–
Log-Indoxyl sulfate (μg/mL)	0.373	<0.001*	0.217	0.133	0.003*

Data of triglyceride, glucose, BUN, creatinine, and indoxyl sulfate levels showed skewed distribution and therefore were log-transformed before analysis.

Analysis of data was done by univariate or multivariate stepwise linear regression analysis (adapted factors were diabetes mellitus, age, SBP, eGFR, and log-indoxyl sulfate).

SBP: systolic blood pressure; DBP: diastolic blood pressure; TCH: total cholesterol; BUN: blood urea nitrogen; eGFR: estimated glomerular filtration rate.

*p < 0.05 was considered statistically significant.

**Figure 1.**
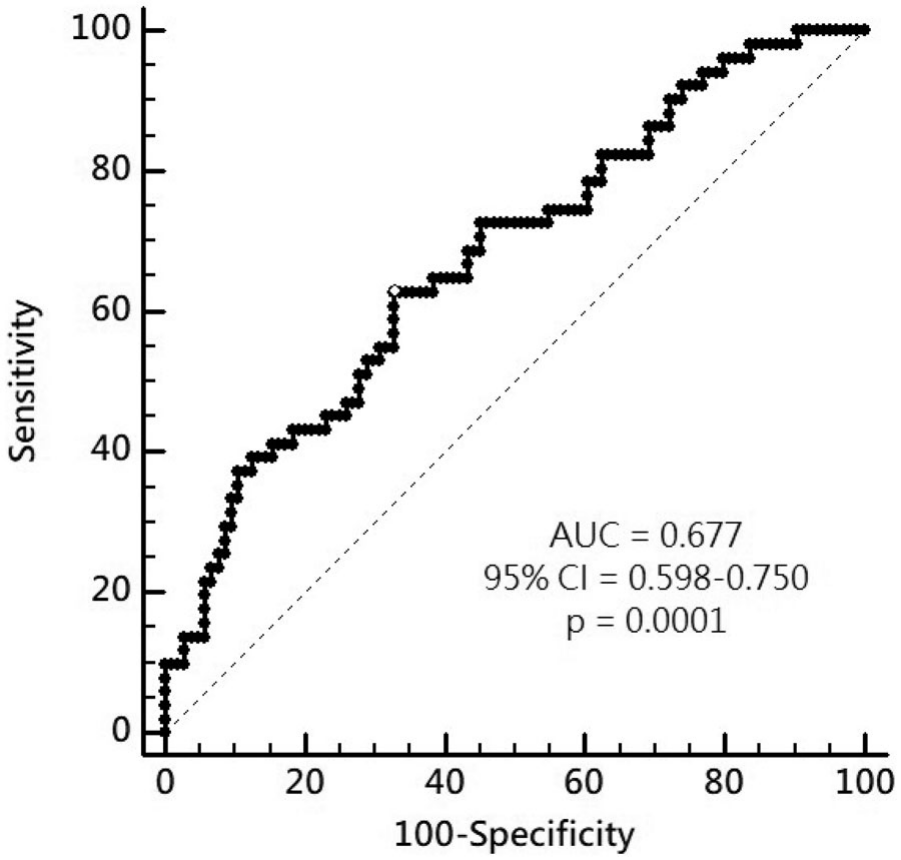
ROC curve and cutoff level of IS to predict AS of CKD patients.

**Figure 2.**
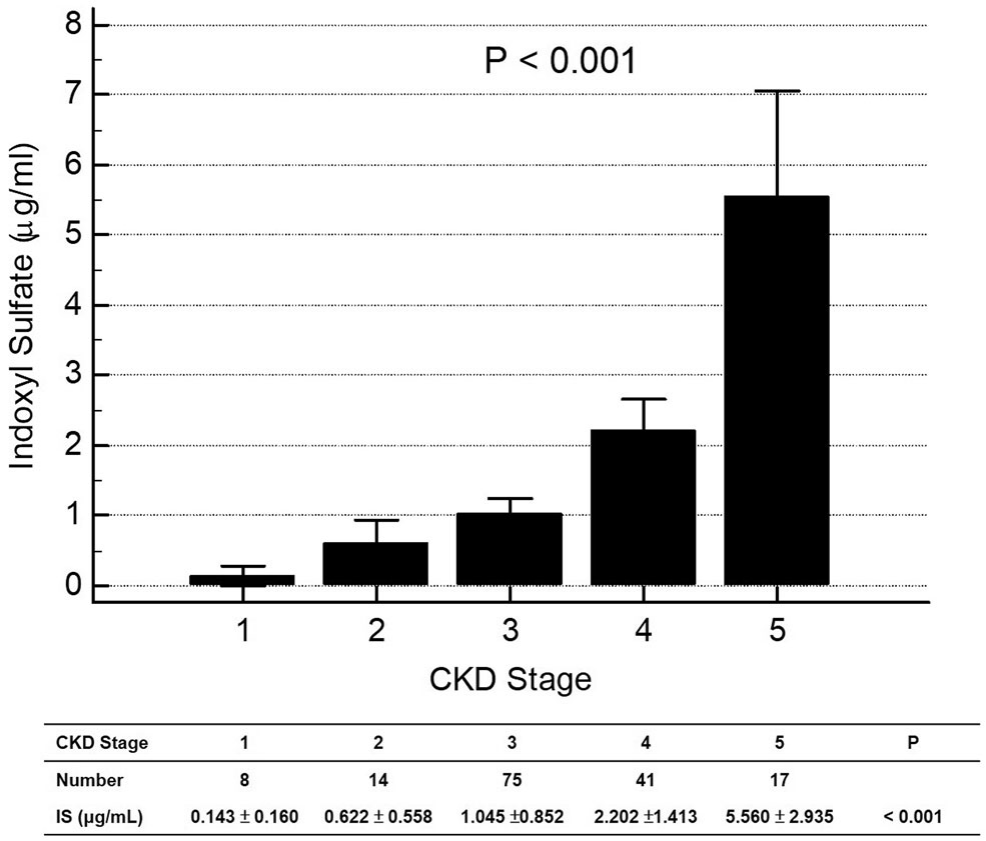
Values of indoxyl sulfate in different stage of chronic kidney disease.

**Figure 3.**
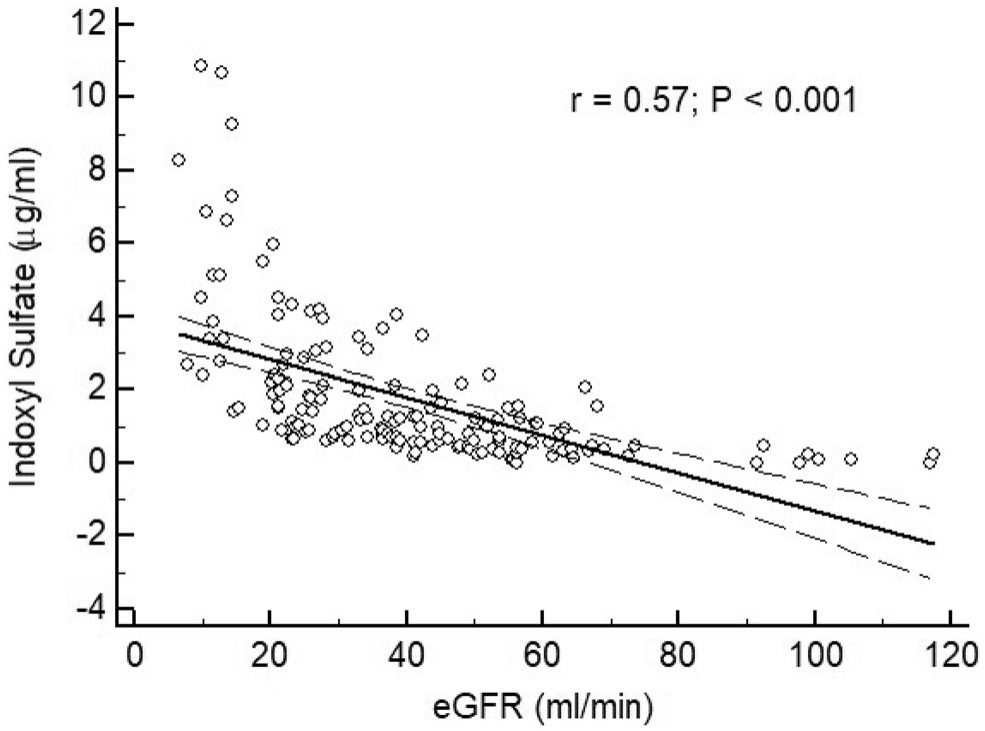
Correlation between values of indoxyl sulfate and estimated glomeural filtration rate of patients.

By analysis of ROC curve, the optimal value of IS to predict AS was 1.227 μg/mL (Youden index 0.3005) with AUC as 0.677 (95%CI 0.598–0.750, *p* = 0.0001), with a sensitivity of 62.75% and specificity of 67.31% (Figure 1). Figure 2 showed progressively significant higher values of IS as renal function worsened. In addition, there was significant negative correlation between values of IS and eGFR of patients (Figure 3).

## Discussion

In this cross-sectional study including 155 patients with CKD, we found that, in addition to old age and hypertension, higher serum IS levels were also positively associated with cfPWV, suggesting its utility as a significant predictor of AS in patients with CKD.

AS is caused by irreversible anatomical changes in vascular wall structures, accompanied with functional abnormalities and increased mechanical strain to low-impedance circulation to vital organs, which subsequently result in renal dysfunction, CVD, and mortality [3,4]. A study of 16,867 European subjects revealed that cfPWV at any age was associated with BP elevation, which indicated that high BP mutually contributed to increased cfPWV [5]. Aging can indeed lead to marked changes in the mechanical and geographic characteristics of the vasculature. However, aged-related changes in stiffness gradient is accelerated in patients with end-stage renal disease (ESRD) and studies have shown a significantly steeper correlation between age and aortic pulse wave velocity (PWV) in association with mechanisms leading to elastin fragmentation and calcification of the medial layer [3,6]. Moreover, studies demonstrated a negative association between renal function and PWV in patients with coronary arterial disease and stage 3 or 4 CKD [7,8]. On the other hand, a meta-analysis revealed that every PWV standard deviation increased was associated with 8% higher risk of incident CKD [21], indicating that vascular stiffness might lead to renal dysfunction. The decreased vascular lumen diameter with the progression of vascular stiffness results in increased pulse pressure and SBP as well as decreased DBP, along with microvascular dysfunction and increased all-cause and cardiovascular mortality [22]. In patients with hypertension with normal renal function, DM with normal renal function, or end stage renal disease patients, studies reported that both BP and age could positively predict cfPWV [17,23,24]. Moreover, in a systematic review on risk factors associated with cfPWV, Cecelja et al. found that age and BP were associated with cfPWV in 91% and 90% of the analyzed studies, respectively; however, only 52% of the studies reported a positive association between DM and cfPWV [9]. In addition, other studies showed that poor glucose tolerance, higher advanced glycation end products, and DM duration were independently associated with central AS, independent of age, sex, and BP [25–27]. Consistent with these studies, we found that old age, higher SBP, impaired renal function, and DM were associated with higher cfPWV. Furthermore, after adjusting for these factors, old age and SBP remained significant predictors of AS in patients with CKD.

IS, which is metabolized in the liver from indole, a product of tryptophan that is produced by intestinal bacterial flora, accumulates with the deterioration of renal function and contributes to the progression of CKD [28,29]; IS is also a marker of poor long-term outcomes in patients with CKD and ESRD [29,30]. Moreover, eGFR correlated negatively with PWV in CAD and CKD patients [7,8], IS was as well to induce dysfunction of vascular endothelial and smooth muscle cells [31–33]. *In vitro* studies demonstrated that endothelial cells incubated with IS exhibited impaired proliferation, wound repair, nitric oxide production, and increased cell senescence and oxidative stress, indicating the adverse effects of IS on endothelial function [32,34]. In addition, IS increased the production of reactive oxygen species, as evidenced by increased expression levels of superoxide and peroxynitrite, and reduced the production of nitric oxide through the induction of nicotinamide adenine dinucleotide phosphate (NADPH) oxidase and the inhibition of glutathione, ultimately leading to endothelial cell dysfunction [31,34]. Furthermore, *in vitro* and *in vivo* studies demonstrated that IS-treated human aortic smooth muscle cells could promote aortic calcification and aortic wall thickening and enhance the expression of osteoblast-specific proteins, senescence, and calcification due to oxidative stress resulting from NADPH oxidase upregulation [35,36]. In studies of patients with CKD, baseline IS levels were negatively correlated with vascular reactivity index [15] and positively correlated with cfPWV and aortic calcification; these studies also found that IS was a predictor for overall and cardiovascular mortality [30]. Importantly, an oral sorbent was reported to reduce serum IS levels and oxidative stress *in vitro* [36]. The same oral sorbent, AST-120, was also demonstrated to reduce PWV and increase flow-mediated endothelium-dependent dilatation in patients with CKD not undergoing dialysis; the underlying mechanism included the inhibition of NADPH oxidase and/or mitochondrial respiration, leading to reduced production of reactive oxygen species [33,37,38]. Collectively, these studies suggest that IS-induced oxidative stress is detrimental to vascular structures through the altered functions of endothelial and smooth muscle cells. In the present study, we found that serum IS levels were positively correlated with cfPWV and that serum IS was a significant predictor for the development of AS; these findings highlight the potentially crucial role of IS in the development of AS by mechanisms including the derangement of pro- and anti-oxidative systems in vascular structures. Further investigation is warranted to elucidate the detailed mechanisms underlying these effects.

The present study has several limitations. First, this was a cross-sectional, single-center study including a small number of patients with CKD. Second, inflammatory markers such as C-reactive protein did not measured. Third, differences in dietary habits as well as dietary supplements can impact the colonic microbiome, which can result in fluctuations of serum IS levels; Fourth, the detailed mechanisms of IS to influence the process of AS could not be clearly deduced without *in vitro* studies. Therefore, the causal relationship between serum IS levels and AS should be evaluated with controlled dietary and nutritional supplement intake in prospective studies of larger patient cohorts and *in vitro* studies to clarify the harmful effects of IS on the vasculature.

In the present study, we demonstrated that, in addition to old age and elevated SBP, serum IS might also be a potential biomarker associated with AS in patients with CKD, indicating that IS might mediate the progression of AS; however, the underlying mechanisms require further investigation.
